# P024: Towards science based transmission intervention protocols for norovirus. Norovirus disinfection: how much is enough?

**DOI:** 10.1186/2047-2994-2-S1-P24

**Published:** 2013-06-20

**Authors:** E Duizer, E Tuladhar, M Koopmans, M Zwietering, R Beumer

**Affiliations:** 1 Department for Respiratory and Enteric Virology, Centre for Infectious Diseases Control, RIVM, Bilthoven, Netherlands; 2 Laboratory of Food Microbiology, Wageningen University, Wageningen, Netherlands; 3 Virology, Erasmus Medical Centre, Rotterdam, Netherlands

## Introduction

Viruses are the most common cause of infectious disease acquired in the indoor environment in hospitals, schools and households causing considerable impact on human health. Transmission of enteric and respiratory viruses is assumed to occur predominantly direct from person to person followed by indirect transmission through contaminated fomites/surfaces. The risk of infection resulting from transmission through contaminated surfaces depends on a number of factors, including the level of shedding of infective particles, their stability on surfaces and resistance to decontamination procedures, fractions transmitted per contact and the dose required for infection. Among the enteric viruses, human noroviruses (NoVs) and rotaviruses are most notorious for causing outbreaks of gastroenteritis within hospitals, nursing homes and cruise ships and are significant cause of hospitalization. Besides human NoV and rotavirus, other enteric viruses like enteroviruses and parechoviruses, and respiratory viruses like influenza and adenovirus may also be transmitted through contaminated surfaces.

## Methods

We performed several studies that focused on reducing the transmission via contaminated surfaces. We studied the effect of several cleaning and disinfection procedures, the transfer rates from fingers to hard surfaces and vice versa, and the effects of hand washing and alcohol based hand rubs.

## Conclusion

These quantitative data were used to establish target levels for residual contamination and thus to determine “how much cleaning is enough” and to redraft our norovirus guidelines to control the spread of these viruses in health care settings in a more efficient and practical way.

## Disclosure of interest

None declared

